# Positive mental health of Latin American university professors: A scientific framework for intervention and improvement

**DOI:** 10.1016/j.heliyon.2024.e24813

**Published:** 2024-01-20

**Authors:** Angel Deroncele-Acosta, Angel Olider Rojas-Vistorte, Andresa Sartor-Harada, Oscar Ulloa-Guerra, Rosendo López-Mustelier, Alejandro Cruzata-Martínez

**Affiliations:** a Universidad San Ignacio de Loyola, Lima, Peru; b Universidad Europea Del Atlántico, Spain; c Universidad Internacional de Valencia, Spain; d Universidad de Oriente, Cuba; e Instituto Peruano de Salud Familiar, Lima, Peru

**Keywords:** Mental health, Virtual education, Hybrid classroom, University, Students, Learning, Teaching, COVID-19, Educational innovation, Digital transformation

## Abstract

The post-pandemic stage covid-19 has revealed overloads, ambiguities, and conflicts of teachers in the performance of new roles in hybrid classrooms that demanded an urgent adaptation, this highlighted the need for priority attention to the mental health of teachers, however, there are still insufficient studies that transcend the diagnosis and are committed to establish proposals for improvement. **OBJECTIVE**: This study aims to establish a proposal for the promotion of positive mental health (PMH). **METHODS:** The study was deployed from a qualitative approach; using an ethnomethodological design that allowed studying how teachers create meanings and sense in their work context, an appreciative interview was conducted with an affirmative theme that allowed teachers to expose their experiences that were systematized and processed with ATLAS. ti software. The application of the interview was conducted online through a Google form, during the months of February and March 2023. Three hundred university professors who experienced the pandemic in universities in Brazil, Chile, Colombia, Ecuador, Mexico, and Peru participated, based on a convenience sampling. **RESULTS:** The results of the deductive phase confirmed Lluch's PMH theoretical framework; however, new nuances or variations have been identified, which must be considered in the complex and dynamic nature of each PMH factor. From there, the results of the inductive phase allowed revealing emerging concepts, that is, new categories that would have the function of improving the PMH factors, which is why they have been denominated: dynamizing nuclei. PMH dynamizing nuclei are adjustment to work environment, soft skills, work-family balance, self-motivation, self-efficacy, subjective well-being, proactive strategies, engagement, resilience. **CONCLUSIONS:** Finally, with the results of both phases, the creation of an integrated model was generated, which was evaluated by six experts in a round of feedback, who highlighted the relevance of the findings and offered recommendations that were considered in the study. The new integrated model has revealed an interesting association, since it not only legitimizes the PMH's dynamizing cores, but also informs on which specific factor of the PMH these cores have the greatest impact, which has a high guiding value for intervention and improvement based on focused strategies.

## 1 Introduction

This study was motivated by the value of paying attention to the mental health of teachers in the COVID-19 post-pandemic stage to provide these professionals and institutions with resources that help in the self-management of well-being. Also considered was the imperative for the post-crisis period to be anticipated to preserve the curriculum and continuity of learning [1].

The post-pandemic has led teachers to an unpredictable scenario, with a legitimization of virtual environments and a positioning with much strength of hybrid environments, this has required a readjustment in the work activity that goes through implications for the mental health of teachers who increasingly require positive job development [2].

Teachers also experience confusion and stress because they are often unsure of their obligations and of how to maintain connections with students in a hybrid context, entailing enormous human and technical challenges [2]. Overnight, teachers had to begin creating and managing virtual classrooms, communicating with students and parents through social media, and learning while providing distance education to millions of students affected by school closures around the world because of the pandemic [2,3].

Indeed, teachers need socioemotional support to cope with the additional pressure of teaching in times of crisis [3]. A recent study argues that the pandemic and subsequent public health measures had a negative impact on people's mental health [4]; the authors conclude that mindfulness-based training can effectively mitigate the negative psychological consequences of the pandemic helping restore the well-being of the most vulnerable.

The real impacts that pandemic has had on mental health call for studies that transcend the diagnostic perspective and offer solutions, as teachers and educational institutions themselves need not only “what happens,” but especially “how to deal with it.” Thus, this study promotes a “know how to do,” “know how to be,” and “know how to live together” in this so-called “digital age,” considering that an effect of the pandemic on the future of teaching and learning in higher education is associated with teachers and students’ mental health [5].

### 1.1 Theoretical background

With the COVID-19 pandemic, universities were forced to close their on-site classrooms due to the need for physical distance between people, as one of the main security measures to avoid contagion; in that scenario, the virtual modality offered the possibility of continuing with classes and avoiding physical contact, hence the virtual learning environments had a great boom and positioning, becoming the most developed modality during the pandemic.

With the arrival of the post-pandemic stage, physical classrooms opened their doors again, but undoubtedly virtuality had brought many advantages that could continue to be used in this new normality [6]. Hybrid classrooms thus re-emerged as a peculiar design for the new normal after the COVID-19 pandemic [7]. The hybrid classroom approach allows synchronizing the virtual environment and the face-to-face environment, so that students can attend the physical classroom, while other classmates attend through a virtual platform, establishing the classroom as a physical and/or virtual time and space context of direct and/or mediated, synchronous and/or asynchronous communication between teachers and students [8].

Teaching in hybrid classrooms has brought challenges and opportunities, particularly for classes with many students enrolled [9]. Synchronizing physical and virtual environments requires addressing economic, social, cultural, environmental and especially human challenges; it may require economic investments to ensure the required quality of platforms such as Hyflex classrooms, a necessary investment if it is properly leveraged because there is already scientific evidence showing how the Hyflex technology-based learning approach is significantly effective in the professional academic performance and attitudes of teachers in training compared to traditional teaching [10].

This adaptation process requires especially a cultural change around the conception of teaching-learning, the teacher must be more dynamic and versatile, be attentive to what happens in the physical space and virtual space, achieve a class where students feel connected to each other, and pay special attention to the design of activities, resource planning, themes, role management, considering that the hybrid classroom approach needs an integration of data from the virtual field and the live field [11], hence in many occasions the teacher needs to innovate to increase the performance of students in the hybrid classroom and the virtual environment [12] which necessarily requires the integration of ICT to these processes of educational innovation in universities [13], however, several challenges remain, not only associated with the usefulness of technological tools [9], but especially around the emotional intelligence of teachers and their mental health in hybrid education [14].

### 1.2 Psychosocial intervention for positive mental health (PMH) in teachers

Currently, the use of ICTs in educational processes is increasingly frequent, positioning hybrid education as an essential proposal in the coupling of virtual and face-to-face contexts. Thus, the hybrid classroom has been designed to allow participants in physical space and online to interact during class sessions [15], however, there are still challenges to overcome including the promotion of positive mental health in teachers who work in these educational environments.

Positive mental health promotion has been gaining recognition as an important teaching competency. Therefore, it is essential that teachers have adequate mental health literacy [16], In this regard a recent study analyzes the experiences and perceptions about an online training program on positive habits as an e-mental health promotion and prevention strategy, showing a positive experience in the implementation of an e-mental health strategy to address the specific needs of teachers and close the gap in access to mental health promotion and prevention services [17]. However, positive mental health interventions for university faculty facing hybrid classes are still scarce.

Teaching evidence-based self-help methods often represents a viable option for addressing the urgent need for psychological support, when in-person services are suspended. The interventions found to be most effective include self-guided cognitive behavioral therapy, acceptance and commitment therapy, and mindfulness-based interventions [4] all of which support the need to promote positive psychological resources.

A recent study revealed that 89.5 % participants have a low level of PMH [18] demonstrating the urgency of developing routes to enhance the well-being of teachers in a scenario that is shifting toward hybrid education.

Several studies have explored how teachers can provide positive mental health (PHM) to others [[19], [20], [21], [22], [23], [24]], but almost no research has been conducted regarding how they should self-manage their own PMH. Other studies recognize the role of teachers as managers in psychosocial intervention for students [25,26] with insufficient intervention approaches toward teachers themselves.

In line with the above, Rothì and collaborators [24] mention the growing expectation of teachers, who not only act as educators teaching classes, but are also acting more as first-level mental health professionals; however, this must include the self-management of their own health. To do so, teachers should become aware of the need for self-care from a socioemotional education perspective that allows for developing psychosocial resources [18], understanding that “awareness and the development of attitudes are the first important steps towards mental health promotion” (p. 139) [19].

PMH is recognized as “an individual asset that contributes to our well-being, quality of life, and creativity” [27] (p. 2). This implies a salutogenic and covitality approach, which emphasizes the positive core of human beings: dreams, hopes, strengths, aspirations, projects, expectations, motivations, leaving the traditional conception of health as the absence of disease behind. In this regard, the World Health Organization [28] considers the following:

Mental health is a state of well-being in which a person fulfills their capabilities and can cope with the normal stress of life, to work productively, and to contribute to their community. In this positive sense, mental health is the foundation of individual well-being and effective community functioning (p.1).

Two theories dominate the field with respect to PMH components: the hedonic approach and the eudaimonic approach. The hedonic tradition is concerned with positive affect (or positive emotions and moods) and high life satisfaction, while the eudaimonic tradition focuses on an individual's optimal functioning in daily life [29].

These approaches: 1) salutogenic, 2) covitality, 3) hedonic, and 4) eudaimonic, should be considered part of “mental health interventions at work (for example, stress prevention programs” [28] which will allow teachers to self-manage their PMH.

However, this is a challenge as the PMH subject has been scarcely studied by teachers. Although there are valuable contributions, such as the proposal by Lluch [30], who suggests an understanding of PMH based on the following factors: Personal Satisfaction (F1); Prosocial Attitude (F2); Self-control (F3); Autonomy (F4); Problem-Solving and Self-Actualization (F5); Interpersonal Relationship Skills (F6), “the measurement of the PMH construct is still incipient” [31] (p. 103). This calls for further deepening of the epistemic mapping of PMH with the commitment to offer theoretical–practical contributions increasingly contextualized to the working conditions and demands of teachers and the personal-work link of university faculty.

This study aimed to identify emerging factors of positive mental health and to design a viable proposal for its psychosocial management. In this sense, the study, which is based on positive psychology, did not direct its attention to the negative or deficit-based aspects; on the contrary, it focused on amplifying the positive core of the participating actors, hence the intention to rescue positive, encouraging, and optimistic projections about mental health in hybrid environments.

## 2 Methodology

### 2.1 Approach and method

The qualitative approach employed was ethnomethodology [32], a design that examined how individuals create meaning in their daily professional performance and attribute meaning to their professional experiences within a specific work context, from an organizational culture. The methodology captured both formal and informal practices, revealing social dynamics and providing a holistic perspective of the teaching experience. In addition, emphasis was placed on individual subjectivity, facilitating teacher participation in the research process. This design produced a rich and contextualized understanding of the positive mental health of university teachers in Latin America, including proactive aspects aimed at improving their PMH, from the different forms of reflection in the experience narratives [33].

As stated, the main methodological design of the study is ethnomethodology, and although it does not directly deal with systematizing experiences in a formal way (as the method of systematization of experiences); ethnomethodology has been consolidated as a useful tool to rescue experiential narratives of teachers in their daily professional practices, and how teachers construct and give meaning and sense in their daily interactions. The application of this approach to the educational setting allowed us to gain valuable insights into the experience of teachers in their work contexts, as we recovered authentic and detailed perspectives on the challenges, strategies and social dynamics that influence educational practices.

While ethnomethodology does not primarily aim to systematize experiences in a structured way, it does draw on teachers' professional experiences to contribute to a detailed understanding of how teachers create and maintain meaning in their concrete socio-educational interactions. In our study, ethnomethodology highlighted five key elements that distinguish it from other qualitative methods.

1. ***Focus on everyday teaching practice:*** The focus of the experiential narratives was on teachers' everyday practices and how they make sense of their world in specific social situations. This allowed for a detailed understanding of real social interactions and the ways in which meaning is constructed on a day-to-day basis.
2. ***Emphasis on practical methods and unwritten rules:*** The importance of practical methods and unwritten rules that guide social behavior was highlighted, allowing us to reveal patterns and norms that may not be evident through other research methods. This allowed us to access positive cores of teachers that were not always conscious, and from there to uncover tacit elements and express them explicitly in the resulting model.
3. ***Development of theory from action:*** This design allowed us to recreate and generate a scientific framework for intervention and improvement of positive mental health. Ethnomethodology allowed us to develop theory directly from social action, rather than applying pre-existing theories to experiences.
4. ***Cultural sensitivity***: Ethnomethodology allowed us a more sensitive approach to cultural and contextual differences, as it allowed us to focus on detailed understanding of specific teaching practices in particular situations.
5. ***Recognition of complexity:*** Finally, ethnomethodology allowed us to recognize the inherent complexity of social interactions and to explore that complexity rather than simplify it, leading to a richer and more detailed understanding of contextualized teaching experiences.

### 2.2 Sample

300 university professors from six Latin American countries participated: Brazil, Colombia, Ecuador, Chile, Mexico, and Peru. The selection criteria for the participants were: 1. willingness to participate on a voluntary basis, 2. having had teaching experience during the covid 19 pandemic, 3. working in hybrid environments in the post-pandemic stage. Of these, 157 were women and 143 men; 129 from public universities, 133 from private universities, and 23 worked in both types of institutions. As for the type of contract, hourly (103), part-time (53) and full-time (144); around age, 174 professors are in the age range of 43–75 years, while 126 professors are in the range of 23–42 years. Finally, in terms of work experience, we have the following distribution: 1–10 years of experience (118 teachers), 11–20 years of experience (124), 21–30 (38); 31–40 (15) and 41–50 (5). As an additional question for characterization, participants were asked to select the study modality that caused them the most stress, according to their perception; they could choose between a) face-to-face, b) virtual and c) hybrid; 74 % mentioned that hybrid classes were more complex and involved more stress. These details are summarized in Table 1.

**Table 1:** Sample characteristics.

Category	Frequency	Percentage
**Gender**		
Female	157	52.3 %
Male	143	47.7 %
**University type**		
Public	129	43.0 %
Private	133	44.3 %
Both	23	7.7 %
**Contract Type**		
Hourly	103	34.3 %
Part-time	53	17.7 %
Full-time	144	48.0 %
**Age Range**		
23–42 years	126	42.0 %
43–75 years	174	58.0 %
**Work experience**		
1–10 years	118	39.3 %
11–20 years	124	41.3 %
21–30 years	38	12.7 %
31–40 years	15	5.0 %
41–50 years	5	1.7 %

### 2.3 Data collection instrument

An interview based on an appreciative inquiry approach [34] was used to analyze the positive mental health category; the interview was based on an affirmative topic that allowed the teacher to make constructive proposals and reveal a positive core that allowed establishing an agenda for positive change. The affirmative topic consisted of mentioning three new challenges for the teacher in hybrid teaching for a successful and healthy performance. All this formed the basis for redefining the configurations and dimensions of the proposed model. Lluchs' multifactorial model of the PMH was adopted to identify the codes associated with the factors of the model: 1.- personal satisfaction factor, 2.- prosocial attitude factor, 3.- self-control factor, 4.- autonomy factor, 5.- problem-solving and self-actualization factor, and 6.- interpersonal relationship skills factor [30].

Based on the above, the positive core of appreciative inquiry was the PMH, while the affirmative topic was consolidated into a single question: “What are the challenges and opportunities that you identify in this digital era for a successful and healthy performance in your profession as a teacher?", around this open question teachers were able to express a wide range of concerns, good practices and projections that allowed, after a rigorous planning process, to build an agenda for positive change [34].

### 2.4 Data management and processing

The interview responses were processed with ATLAS. ti version 8 software, based on content analysis to interpret the subjects' experiences. This made it possible to extract discursive codes and group them into emergent categories. ATLAS. ti was used to organize, categorize, and analyze qualitative data in a structured approach, facilitating the identification of patterns, the generation of new concepts and the construction of models in the context of PMH promotion. The interviews generated a large amount of information, so the software's ability to group codes was fundamental to identify dynamizing nuclei and understand the relationships between the key elements of the study. The ATLAS. ti software was used in two phases, a deductive phase, and an inductive phase.

#### 2.4.1 Deductive phase

We proceeded to import Lluch's PMH theoretical framework into ATLAS. ti, identifying the predefined factors and categories, then proceeded to code the data according to the predefined categories of the theoretical framework, to analyze how the data align with the existing categories and if there are new nuances or variations not considered in the theoretical framework, to finally move to the examination of patterns and relationships between the existing categories, identifying any unexpected variations or relationships.

#### 2.4.2 Inductive phase

It began with the importing of the literal transcription of the interview to initiate the process of generating emerging codes. This new round of inductive coding in ATLAS. ti was essential to identify patterns, themes and emerging concepts that were not initially contemplated in the theoretical framework. This was then followed by the process of clustering and categorization, grouping the new inductive codes in a way that reflects the emerging categories, this indisputably allows for adjusting the existing categories to accommodate the new findings, and finally concludes with the process of creating an integrated model.

In this way, ATLAS. ti allowed us to organize the teachers' responses based on the PMH theoretical framework, while at the same time allowing us a more proactive perspective to find PMH dynamizing factors by visualizing and organizing both deductive and inductive categories in an integrated model. This approach allowed us to leverage the strengths of both logics, integrating the structure provided by the theoretical framework with the flexibility to discover and adapt to new and emerging categories. ATLAS. ti facilitated the coding, clustering, and visualization process during both phases of the analysis. In addition, the findings were shared with six experts (one for each participating country) who provided recommendations that were considered in the study. The participation of an expert from each country was interesting and valuable, as it contributed to align cultural and contextual aspects, being a moment of reflection and agreements.

## 3 Results

For this qualitative study, ATLAS. ti was used to systematize the open questions (affirmative topic, constructive propositions), with which categorization and coding were established, revealing groups of codes associated with emerging categories that became essential processes for the proposed model.

The qualitative phase had two stages: Stage 1: Identify codes related to the PMH. To do so, this study assumed the PMH precepts by Lluch [30] and Lluch and collaborators [35] Stage 2: Generate new codes that can be used to reveal emerging categories that constitute psychosocial well-being factors for enhancing the PMH, Salanova, Llorens, and Martínez cited in Ref. [18]. This was used to establish a model that transcends the perspective of evaluating the PMH and offers a dynamic for its development.

It is important to note that Stage 1 involved a deductive logic, factor-code logic (i.e., codes were identified from the PMH factors established by Lluch), and Stage 2 deployed an inductive logic or code-factor logic (codes of the same nature were grouped together and, from this, emerging categories were revealed).

Lluch and collaborators [35] provide six factors for PMH: the **personal satisfaction factor** is related to the elements of the following: self-concept, self-esteem, satisfaction with personal life, and an optimistic view of the future; the **prosocial attitude factor is associated with** active predisposition toward society, altruistic social attitude, helping/supportive attitude, acceptance of others, and differences in social characteristics. The **self-control factor** refers to the ability to cope with stress/conflict situations, emotional balance/control, tolerance to frustration, anxiety, and stress, whereas the **autonomy factor** is related to one's own standards, independence; self-regulation of one's behavior, and sense of personal security/self-confidence. Another factor is **problem-solving and self-actualization**, associated with the analytical capacity, decision-making skills, flexibility, ability to adapt to change, and attitude of continuous growth and personal development. The **interpersonal relationship skills factor** refers to the ability to establish interpersonal relationships, empathy/ability to understand the feelings of others, ability to provide emotional support, ability to establish and maintain close interpersonal relationships. The codes associated with each of these factors are shown (Table 2).

**Table 2:** First stage: Codes related to the PMH.

PMH factors	Codes
Personal satisfaction	honesty: openness to change, work-life harmony, healthy eating, taking on new educational challenges; physical activity, taking care of one's vision and posture; research, innovative, and enterprising nature; training and updating, willingness to improve, adaptability, openness, and trust; culture of respect, collective commitment.
Prosocial attitude	humanization of teaching; acceptance of cultural diversity in the classroom; fair treatment for each student; helpful, understanding, and docile attitude, understanding and managing student support; communication with the family; accompanying the student in virtual environments; empathy, dynamism, and solidarity; inclusive education; use of new tools, flexibility with evaluation; understanding the socioemotional aspects.
Self-control	resilience^a^; healthcare^a^; stress management^a^; emotion management^a^; time management; coping positively with crises; preparing for challenges; tolerance with the pace of work.
Autonomy	adjustment to change^a^; resilience and creativity; motivation; teamwork; daily learning, patience and tolerance in life, adaptation, creativity, and innovation; researching, unlearning, and adjusting; sustained motivation.
Troubleshooting and Self-actualization	research^a^; digital competence^a^; innovation of pedagogical practice^a^; ICT update; adjustment to change^a^; being innovative, strategic, and a leader; updating, renewal and learning; online evaluations, continual training, motivation, and creativity; ability to transmit knowledge; the change of mentality; student motivation; sharing experiences, learning to learn.
Interpersonal Relationship Skills	empathy****; empathy with students; teamwork in virtual environments; communication skills; active learning methods; soft skills for remote work; empathy; calm; shared responsibility; enthusiasm at work; dedication and love of work; achieving students' interest; research teams; encouraging proactivity in students.

a A high frequency of this code is observed throughout the data. The prevalence of this code stands out compared to others in the same code network.

From an appreciative inquiry approach that emphasizes positive aspects and strengths, these results show that teachers' positive mental health was characterized by a balanced combination of factors that encompass both personal and professional dimensions. The findings reflect that teachers with positive mental health exhibit personal satisfaction derived from their openness to change, work-life balance, healthy habits, and willingness to take on educational challenges. In addition, they excel in prosocial attitudes, demonstrating empathy, acceptance of cultural diversity and fair treatment of each student. Self-control is fundamental, evidenced by resilience, effective stress management and attention to physical and emotional well-being, as well as autonomy, which is manifested in adaptability to change, creativity, motivation, and effective collaboration in teams. While problem solving and self-actualization are reflected in active participation in research, digital competence, pedagogical innovation, and constant updating. Finally, interpersonal relationship skills, based on empathy, effective communication, and the promotion of proactivity, are key to maintaining healthy relationships with students and colleagues.

Although the results reveal several positive elements associated with teachers' mental health, there is some dispersion in their presentation. The diversity of factors, although beneficial, suggests the need for a deeper review and a cohesive interpretation to build an organized system, so we move to the second stage of analysis, to identify interrelationships and hierarchize key elements. This would facilitate the implementation of a specific and personalized proposal, thus promoting sustainable wellbeing in the educational environment.

As a result of the dispersion in the presentation of elements associated with teachers' positive mental health, there is a pressing need to move towards a proactive projection stage. Beyond the diagnosis and identification of relevant aspects, this new phase seeks to identify dynamizing nuclei that act as fundamental drivers of positive mental health. By identifying recurrent patterns, key strengths and synergistic relationships between the different codes, new emerging categories were revealed, a superior grouping of all the codes (synthesis); each new category thus became a PMH developer, which then made it possible to establish the proposal with an organized and proactive approach.

Next, we proceed to present the coding and categorization in ATLAS. ti to complete Stage 2 of the qualitative phase, which proves that managing a PMH is a complex process, hence the importance of these emerging categories to structure a theoretical model that serves as a basis for designing future practical interventions. These interventions include strategies, procedures, change agendas, programs, techniques, action systems, manuals, communities of practice, methodological tools, collaborative groups, dialog support tables, and workshops, among others (Table 3).

**Table 3:** Second stage: Emerging categories for enhancing PMH.

Codes	Emerging Categories
pleasant and positive work environment, good pay, teamwork; climate of support and innovation; the creation of attractive and suitable materials; the promotion of digital competence; setting work schedules; adaptability, openness, and trust; good work relations; greater recognition of teaching work; access to internet services.	Adjustment to work environment
self-regulation of emotions, interdisciplinary teamwork, acceptance of diversity; justice and fairness, flexibility in relationships, empathy in virtual work, student support; good communication in digital environments; communicative skills; understanding the socioemotional; non-verbal communication.	Soft skills
work-family balance, enjoying time with family; work organization, raising awareness of work and family roles, “not transfer problems from work to home,” life and work harmonization.	Work-family balance
motivation, creativity, motivation for learning, continuous training, ongoing training and learning, continuous improvement, teamwork; dynamism, discipline, and perseverance; updating, renewal and development in ICT; innovative, strategic, and leading teacher.	Self-motivation
assertive communication, student empathy, student attention and motivation, shared responsibility, effective and affective communication; motivating students with ICT.	Self-efficacy
autonomous learning, pedagogical autonomy, design of motivating environments; professional development; empathy in classes, affective interaction, empathic relationships; ICT humanization; autonomous work with ICT; technological mastery; learning to relax; virtual group work; harmony with the classroom environment; positive management of the environment; soft skills development; purpose, leadership, entrepreneurship, and resilience.	Subjective well-being
attention to workload; stress prevention, self-help resources; mental health management; disruptive process mentality, coping with uncertainty.	Proactive strategies
dedication and love of work, digital culture, ICT appropriation, enthusiasm at work, proactivity, innovative research.	Engagement
resilience, self-control, adaptation to change, stress management, mental and physical health care, coping with uncertainty, performance capacity in changing contexts, emotion management; avoiding overload, stress, optimistic attitude.	Resilience

**Fig. 1:**
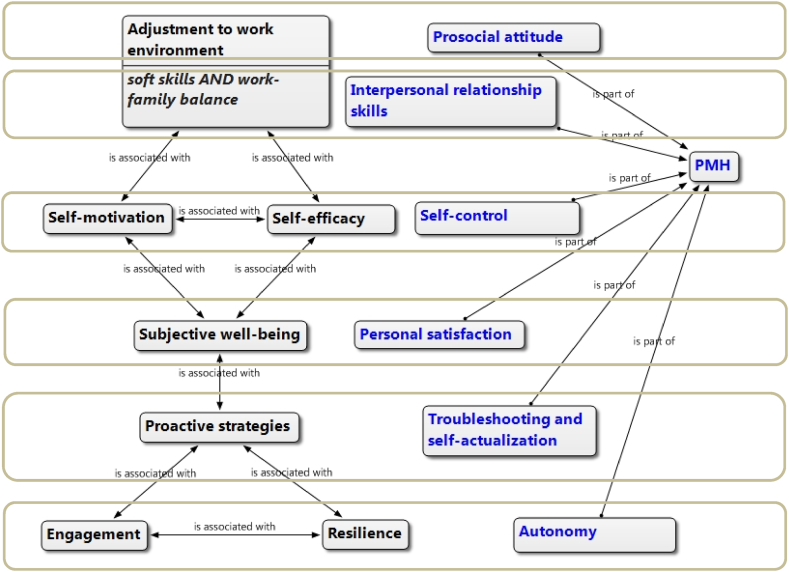
Model of psychosocial intervention in the PMH of teachers.

**Source:** ATLAS.ti (version 8).

These resulting categories were visualized in an ATLAS. ti semantic network that helped to understand the emerging model (Fig. 1); especially from the linking elements offered by the program, such as association, relationship, cause, contradiction, property, subordination, among the categories included [36].

Adequate management of positive mental health requires, as a premise, a good adjustment to the work environment, in which socioemotional skills and the balance between family life and work life have a direct impact. This adjustment to the work environment is also achieved when the subject implements his or her self-efficacy and self-motivation tools, experiencing subjective well-being; this in turn becomes stable and lasting when the worker deploys proactive strategies, by virtue of being more engaged and resilient.

The new integrated model has revealed an interesting association, since not only are PMH dynamizing nuclei identified, but it is also possible to verify which specific PMH factor has greater emphasis on each nucleus and its system of relationships. This has an important interventive value to achieve focused strategies; thus, prosocial attitudes can be developed with greater emphasis from the adjustment to the work environment, while the factor of interpersonal relationship skills would be promoted especially from soft skills, self-control finds its main energizer in self-motivation and self-efficacy, in the personal satisfaction factor, subjective well-being has a greater incidence, while self-actualization would be promoted more directly from proactive strategies. Finally, the PMH autonomy factor is achieved more precisely through resilience and engagement.

As we can see, this model is holistic, so that these relationships are not linear or unidirectional; on the contrary, it is a complex framework in which all its components presuppose and complement each other; nevertheless, it is considered that these findings of association between dynamizing nuclei and PMH factors have a high guiding value.

A round with six experts was held for the presentation and discussion of results, which took place in a 2-h meeting via ZOOM; in general, all experts validated the relevance of the findings, highlighting as a relevant and novel aspect the integration of the inductive and deductive phase as it became a guiding and clear tool to manage positive mental health.

## 4 Discussion

### 4.1 Why a proposal for psychosocial mental health management of teachers in a post-pandemic period?

Hurtado et al. [37] argue that “in order to be able to develop interventions aimed at promoting PMH, it is necessary to have conceptual models to guide the actions to follow” (p. 6), as “much research has focused on contextual factors that influence implementation, but they have less attempted to provide an integrated understanding of the mechanisms” [22] (p. 665).

In the same way, we seek to complement what has been suggested by Ref. [29] who proposed the relevance of understanding PMH from internal factors (e.g., emotional, and psychological) of PMH in relation to external factors (e.g., social support, association). Hence, this model is based on its psychosocial nature, with one dimension placing greater emphasis on psychological aspects and another dimension on labor-social aspects.

The participation of teachers is significant; specifically [38], state that “the success of school-based mental health interventions is often inconsistent, in part due to the lack of teacher participation” (p. 312). Programs that do not necessarily reflect the reality of teachers are often achieved, which is why a constructive proposition journey was deemed an element of appreciative inquiry in this study [34] taking the form of open propositions from teachers based on an affirmative topic (qualitative question). Thus, this model is the result of how teachers construct and maintain meaning in their day-to-day professional interactions and experiences.

The feeling that the pandemic passed may be dangerous in the sense of a legacy of inactivity, when in fact the repercussions of the pandemic are being experienced today, especially in the field of mental health, which calls for greater concern and action by the helping professions and institutional and social mental health policies. Specifically, the impact of the pandemic on teachers’ mental health has been an important issue requiring responses [39,40]. This is demonstrated by a study that analyzed the development of psychiatric pathologies/burnout syndrome and its possible risk factors in teachers in the context of the COVID-19 pandemic [41], the same study that calls for prospective research to improve the mapping of this situation, while other authors emphasize that “with the emergence of works investigating the educational impacts of the COVID-19 pandemic, empirical studies assessing the mental health of teachers during the pandemic have been scarce” [42] (p.593).

There are still few scientific findings that relate the variables digital competencies, engagement, and role stress with the positive mental health of teachers. Directly, a recent study that examines the relationship between these three variables concludes on the need to articulate proposals to manage positive mental health in university teachers [43]. Another study examining work-related stressors, including role ambiguity and role conflict, concludes that they are related to psychological maladjustment and poor mental health, while role ambiguity and role conflict (aspects of role stress) were positively related to symptoms of depression, anxiety and stress; thus the study provides preliminary evidence suggesting the development of integrative models of mental health that consider work-related stressors together with personal resources such as emotional resources with the aim of preventing poor mental health in teachers [44].

Another important study with the aim of examining whether (1) role reward (within and between roles) directly contributes to mental health and buffers the negative impact of role stress and (2) the associations between role occupation, role stress and role reward and mental health vary by race/ethnicity, concluded that role reward buffers the negative impact of stress on social functioning [45] which is aligned to the positive mental health psychosocial management approach designed in the present research.

Also, one of the models widely spread in the scientific field indicates how the work experience indirectly influences mental health [46] hence the importance of the proposal of a contextualized socio-labor management that requires self-motivation at work, an understanding of work self-efficacy, a projection of the adjustment to the work environment to finally achieve the construction of subjective well-being.

The authors Kelloway y Barling argue that the characteristics of the job and the stressors of the role, justify and encourage further research on the effects of job design on the mental health of workers in the workplace. With this model they identified job and role characteristics that predict work-related mental health, work-related affective well-being (i.e., job satisfaction, emotional exhaustion, and depersonalization), and subjective competence (i.e., personal accomplishment at work) [46].

On the other hand, there is more scientific evidence on the impact of engagement on mental health and although these studies on teachers are scarce, there is sufficient evidence to demonstrate the importance of engagement as a driver of mental health. In this regard, current studies in digital, rural, and other settings ratify this line [[47], [48], [49], [50]] this is further legitimized by the results of one of the most cited studies that found that educational success and mental health vary according to school engagement profiles [51]. Given all that has been raised and the impact of a COVID-19 scenario, recent studies acknowledge the absence of interventions so they argue that the pandemic could be associated with a legacy of depression, particularly in settings with limited mental health support services [52]. Consistent with this approach authors Tomlinson and Marlow [53] who argue:

“As important as the papers in this special issue are in highlighting the impact of the pandemic on mental health, perhaps their greatest value is in showing how little of what is known has been acted. Epidemiological studies often run the risk of “admiring the problem,” of merely describing the social determining of health” (p.1)

For this reason, this research goes beyond the contemplative frontier and proposes a way of approaching positive mental health as a tool for institutions and professionals to guide their policies and actions in this regard.

### 4.2 Model of psychosocial intervention in the positive mental health of teachers

For the psychosocial management of PMH in the organizational context, it is necessary to start with self-motivation at work, constituted in an understanding of the subject in the integration of creative self-efficacy, motivation for self-learning, self-confidence, and psychological enhancement [54]. This process occurs in relation to understanding work self-efficacy as a process that indicates the capacity perceived by teachers in managing tasks, managing emotions at work and their behavior in the social sphere, achieving a critical understanding, both in the defense of their own viewpoint (assertiveness) and in understanding the states and needs of others (empathy) [55]. These interactions of work self-efficacy must be understood by teachers to make constructive decisions that support their mental health.

This dialectical relationship between self-motivation at work and understanding of work self-efficacy takes shape in and from a projection of adjustment to the work environment by the teacher, which indicates (a) the professional recognition by the teacher, and (b) constructive work environment [56] so institutional proactivity associated with achieving a positive socio-psychological climate is important [18], where teaching work is cared for, recognized, and encouraged, while the teachers themselves as active beings must be able to participate in the organizational projection. Here, the subject-group-organization link plays an important role, if it is a positivity link, then the relationship process between self-motivation at work, work self-efficacy, and adjustment to the work environment, allows for constructing subjective well-being. The latter will be more successful in linking teachers to continuing social-emotional education [18] that ensures effective subjective resources. This framework expresses the dimension of contextualized social-work management.

Subjective well-being is understood from the integration of autonomy, environmental mastery, personal growth, self-acceptance, positive relationships with others, and purpose in life. Positivity is an essential core [57] used to enhance the positive elements of mental health; therefore, the construction of subjective well-being connects the two dimensions of the model.

This means that the contextualized social-work elements of mental health are important but not sufficient to promote PMH, since it requires the integration of effective subjective resources such as proactive strategies, which refer to strategies of the subject regarding 1) their self-regulation (ability to identify and use resources to deal with stress factors) and 2) co-regulation (ability to seek and receive social support from colleagues) [56].

The strategies learned and used by teachers differ in terms of reactivity and proactivity. Reactivity manifests itself when a potentially stressful situation has already occurred; teachers may react with reactive coping strategies by regulating their feelings and emotions so that stress decreases. For their part, proactive strategies (both self-regulated and co-regulated) involve preventing or acting preemptively to mitigate potentially stressful events, thus neutralizing the stressor before it becomes harmful [56] which is also known as effective management (prevention and/or control) of role stress [58].

Thus, teachers must achieve an internalization of proactive strategies. Internalization is the intra-psychological reconstruction of an inter-psychological operation through signs, being an active process of interaction and personal reconstruction. As a result, the personal reconstruction of these proactive strategies requires a professional commitment. In this regard, scientific evidence in a recent multi-country study shows that if teachers increase their levels of engagement (vigor, dedication, and absorption), role stress decreases, highlighting the protective capacity of engagement [43]. Therefore, it requires an engagement invigoration as a process that intensifies the subject's experience, allowing him/her to be attentive to what happens in their context, even helping project the adjustment of the work environment. Therefore, it is presupposed and complemented with the understanding of proactive strategies.

Engagement is considered a positive factor in the organizational context [59]. In this regard, scientific evidence shows that engagement is a driver of teachers' well-being and positivity [57] and that it is possible through proactive strategies to achieve a direct link between engagement and subjective happiness [56].

Finally, this relationship between the internalization of proactive strategies and engagement invigoration leads to the appropriation of a resilient culture based on psychosocial well-being factors, in a subjectivation–objectivation process of mental health, where resilience acquires significance and meaning for the person, and mobilizes their behavior. An example at a practical level is found in people who can reflect on stressors, and their perception of risk helps them be more careful with their mental health. In this study, the resilience model for adults is assumed, comprised five dimensions: internal control, coping style, optimism, personality preference and acceptability [60]. This is a specific proposal of resilience in the mental health of teachers, which requires literacy, with awareness and socioemotional education allowing for appropriating a culture of resilience based on factors of psychosocial well-being.

In summary, the model is established on the basis of two dimensions: a contextualized social-work management dimension resulting from the interactions between the factors: self-motivation at work, understanding of work self-efficacy, projection of adjustment to the work environment and construction of subjective well-being, and a meta-reflexive psychological management dimension because of the interaction between the following factors: construction of subjective well-being, internalization of proactive strategies, engagement invigoration, and Appropriation of a resilient culture based on factors of psychosocial well-being. Finally, the dialectical relationship between both dimensions takes form in the psychosocial management of positive mental health link.

In agreement with Ganga and Kutty [27] although there is conceptual research on PMH, most of the measures capture only some elements and pay very little attention to their multidimensionality. Considering the above and recognizing PMH as a complex and dynamic psychosocial process, the model presented can enrich the multifactor model of positive mental health [30] by integrating other factors. In any case, this study does not intend to establish a new scale, but rather to offer a route to enhance PMH as still insufficient proposals allow for its development based on factors of psychosocial well-being and self-control [18].

However, while the importance and significance of Llul's (1999) PMH scale is recognized, having several negative items can reinforce the deficit, so a more focused view is needed to amplify the positive core of people. This, to transcend the change based on the deficit to a perspective of positive change [34], with emphasis on the subject's possibilities and the strengths of the human being [37,61], considering positive psychological states as protective factors of physical and mental health [62].

## 5 Implications at the educational level, with emphasis on hybrid education

The analysis of the model confirms that adjustment to the work environment is an indispensable premise for teachers to adapt to the hybrid context and their healthy performance, as well as the basis for achieving greater clarity in their roles and adequacy of their workload [58]. This, especially in a new hybrid-training context that involves using digital competence with critical criteria, understanding its pedagogical meaning, beyond the practical functionality of the tools.

Thus, hybrid education involves establishing tools that allow for meta-regulated learning [15]. involving a challenge for teachers who must boost interactive didactic resources to interconnect the thinking and action of students who coexist, some under a face-to-face modality and others virtually. A viable resource can be found in TPACK as integration between technology, pedagogy, and knowledge, a model that has demonstrated its effectiveness for the reflexive integration of technology in the teaching-learning process [63].

The findings from this study allow us to revive possibilities such as the high level of engagement (teachers’ commitment) due to the positive impact demonstrated in the development of digital competence [43]. In this sense, the model establishes the engagement invigoration, but it is possible with the internalization of proactive strategies; thus, teachers should be more open to seek and receive social support from colleagues for a successful and healthy professional performance.

Considering that hybrid education is considered a shift in the educational paradigm [64]. this implies that teachers must be prepared to assume new forms of pedagogical actions, also involving an analysis of educational data on the current state of university learning to generate an effective transition to the hybrid educational model [65]. Therefore, self-motivation at work is vital here, especially concerning creative self-efficacy, and work self-efficacy, emphasizing not only task management but also emotion management.

One factor that promotes this integrated management of tasks and emotions is time management. It is valid to point out that there is a significant but inverse relationship between time management and work stress [66] so the better time management is achieved, the less stress teachers will feel, and this will have a favorable impact on their PMH and performance.

Programs that link online and hybrid education are already available [67] and it is impossible to force students to remain in a classroom in person because certainly the risk of new pandemic outbreaks or infections persists, and the resistance and fear of families is natural, although they are assimilating increasingly more attitudes of self-care to minimize these risks.

The transition to the hybrid modality also involves infrastructure, economic-financial, material, technological and human resource elements, so ultimately hybrid education will be effective to the extent that institutions and governments contain educational policies aligned with developing hybrid as a process that continues to ensure quality education for all. In addition, to the extent that the main educational actors can adequately manage a successful and healthy professional performance, for which the model is a viable option.

## 6 Conclusions, limitations, and prospects

The various codes identified in relation to PMH of teachers show the complexity and diversity in the approach to this phenomenon, finally integrated in the proposed model, which is made available to the educational and scientific community, especially to teachers and institutions. The psychosocial management model of positive mental health of teachers in hybrid environments has two dimensions: a contextualized social and work management dimension resulting from interactions between the configurations: 1.- self-motivation at work, 2.- understanding of work self-efficacy, 3. - projection of adaptation to the work environment, and a meta-reflective psychological management dimension resulting from the interaction between the configurations: 1.- internalization of proactive strategies, 2.- dynamization of commitment, and 3.- appropriation of a resilient culture based on psychosocial well-being factors; both dimensions have a common configuration that connects them, namely the construction of subjective well-being.

The effective development of this proposal requires mental health literacy, focused on socioemotional education, and that professionals and institutions become aware of the need to promote PMH in an educational context that is moving towards a hybrid model as a more permanent solution. For a long time, teachers have been seen more from their role as managers of the mental health of other individuals, but teachers are also required to take care of their own health and institutions and decision-makers are increasingly aware of this. This study could be extended to a larger sample of teachers and include other countries in the Latin American region and worldwide. Similarly, prospective longitudinal studies can be projected to verify the relevance, optimization, functionality, and impact of the proposal.

Among the main limitations of this study we find the size of the sample, taking into account that 300 teachers do not constitute a representative sample related to the population of university teachers in Brazil, Chile, Colombia, Ecuador, Mexico, and Peru, which would be limiting the possibility of a greater generalization, in any case it is an intentional non-probabilistic sampling that required the use of qualitative techniques that allowed deepening the construction of meanings and meanings of the participants. Another limitation of the study is that although a design and validation of the proposed model was achieved, it has not yet been possible to apply it through strategies, programs, actions; Therefore, it is convened that subsequent studies can apply this proposal at a practical level, thus longitudinal studies are projected that allow evaluating the evolution of positive mental health before and after the application of the proposal, so that its relevance can be corroborated, functionality and impact. It would be interesting to conduct longitudinal studies that can compare the levels of mental health and role stress of teachers in a) face-to-face, b) virtual and c) hybrid modalities.

As raised in an interesting study, although both students and teachers value hybrid virtual classrooms positively, challenges remain around improving social presence, to overcome this, teachers require a better understanding of meaningful learning activities to stimulate interaction and communication between online and face-to-face students [68], a topic that should be analyzed in greater depth is the impact of teleworking technostress on satisfaction, anxiety and performance [69]; related to mental health future studies should focus on positive nuclei for its dynamization as happens for example with coping strategies as mediators of academic stress [70], the relationship between effective personality and occupational health of teachers [71], so that teachers can face happy and motivated to the educational challenges of the post-pandemic stage, which requires rethinking the use of ICT during confinement in higher education [72], and to be open-minded to new changes, from the point of view of a reflective, proactive, flexible teacher, able to adapt to the dizzying contemporary digital transformation by promoting technological innovation processes, from the dynamization of research and knowledge management in and from ICT, digital engagement, and especially the management of socioemotional well-being in the online community [73].

Verification and credibility of the findings, emerging codes and categories have been carried out through a sound methodological approach that integrates both the deductive and inductive phases of data analysis in ATLAS. ti. Data triangulation, expert review and theoretical persistence have been additional strategies to reinforce the verification and credibility of the findings. Thus, consistency between data collected from multiple sources, comments from expert colleagues in each participating country, and continued alignment with underlying theory have contributed to the robustness of our findings. Ultimately, the creation of an integrated model that reflects both predefined and emerging categories demonstrates the ability of our approach to capture the complexity of the phenomenon studied. This meticulous and thoughtful process of continuous verification and adjustment has reinforced the internal validity and credibility of our findings, providing a solid basis for the interpretation and application of the results of this ethnomethodological research.

## Ethics declarations

This study was reviewed and approved by [Education Studies Network], with the approval number: [221,028,219].

All participants provided informed consent to participate in the study.

## Data availability statement

The data that has been used is confidential.

## Funding

This study did not receive funding.

## CRediT authorship contribution statement

**Angel Deroncele-Acosta:** Writing – review & editing, Writing – original draft, Visualization, Validation, Supervision, Software, Project administration, Methodology, Investigation, Formal analysis, Data curation, Conceptualization. **Angel Olider Rojas-Vistorte:** Writing – review & editing, Methodology, Formal analysis, Data curation. **Andresa Sartor-Harada:** Methodology, Investigation, Conceptualization. **Oscar Ulloa-Guerra:** Methodology, Investigation, Conceptualization. **Rosendo López-Mustelier:** Methodology, Investigation, Formal analysis, Conceptualization. **Alejandro Cruzata-Martínez:** Methodology, Investigation, Conceptualization.

## Declaration of competing interest

The authors declare that they have no known competing financial interests or personal relationships that could have appeared to influence the work reported in this paper.

## References

[1] (2020). Unesco, COVID-19 Webinar: A New World for Teachers, Education's Frontline Workers - COVID-19 Education Webinar #2.

[2] Aperribai L., Cortabarria L., Aguirre T., Verche E., Borges Á. (2020). Teacher's physical activity and mental health during lockdown due to the COVID-2019 pandemic. Front. Psychol..

[3] (2020). Unesco, COVID-19 Crisis and Curriculum: Sustaining Quality Outcomes in the Context of Remote Learning.

[4] Matiz A., Fabbro F., Paschetto A., Paolone A.R., Crescentini C. (2020). Positive impact of mindfulness meditation on mental health of female teachers during the COVID-19 outbreak in Italy. Int. J. Environ. Res. Publ. Health.

[5] Pelletier K. (2021). https://bit.ly/3bC6f8J.

[6] Atúncar-Prieto C., Deroncele-Acosta A. (2021). Proceedings - 2021 16th Latin American Conference on Learning Technologies.

[7] Triyason T., Tassanaviboon A., Kanthamanon P. (2020).

[8] Deroncele Acosta A., Gross Tur R., Medina Zuta P. (2021). La autonomía pedagógica como potencialidad formativa en los actores educativos del aula. Revista Conrado.

[9] Roy M., Roy M. (2022). Are the Technological Tools Used in Virtual and Hybrid Classrooms Still Useful in a Fully In-Person Setting? an Assessment of the Effectiveness of the Technological Tools in Enhancing the Pedagogy in the New Normal. ASEE Annual Conference and Exposition, Conference Proceedings.

[10] Amirova A., Zhumabayeva A., Zhunusbekova A., Kalbergenova S., Nygymanova N., Arenova A. (2023). Effect of using Hyflex technology learning on preservice teachers' success and attitudes. Int. J. Educ. Math. Sci. Technol..

[11] Schlosser W.E., Aumell A.J., Kilkenny M.M. (2023). Hybrid classroom approach: virtual and live field data integration. J. Nat. Resour. Life Sci. Educ..

[12] Emami T. (2022). An innovation methodology to increase students performance in hybrid classroom and virtual environment. ASEE Annual Conference and Exposition, Conference Proceedings.

[13] Deroncele-Acosta A., Medina-Zuta P., Goñi-Cruz F.F., Montes-Castillo M.M., Roman-Cao E., Gallegos Santiago E., Innovación E. (2021). Educativa con TIC en Universidades Latinoamericanas: estudio Multi-País. REICE. Revista Iberoamericana Sobre Calidad. Eficacia Y Cambio En Educación.

[14] Leonardo Z.V.L. (2020).

[15] Mollo-Flores M.E., Deroncele-Acosta A. (2021). XVI Latin American Conference on Learning Technologies.

[16] Nalipay M.J.N., Chai C.-S., Jong M.S.-Y., King R.B., Mordeno I.G. (2023). Positive mental health literacy for teachers: adaptation and construct validation. Curr. Psychol..

[17] Cepeda-Torres J.F., Patiño Zapata M., Alzate Montoya M.A. (2023).

[18] Soto-Crofford C., Deroncele-Acosta A. (2021). Salud mental positiva en una comunidad de docentes en Ecuador. Maestro Y Sociedad.

[19] Askell-Williams H., Lawson M.J. (2013). Teachers' knowledge and confidence for promoting positive mental health in primary school communities. Asia Pac. J. Teach. Educ..

[20] Cruz C.M., Lamb M.M., Giri P., Gaynes B.N., Matergia M. (2021). Perceptions, attitudes, and knowledge of teachers serving as mental health lay counselors in a low and middle income country: a mixed methods pragmatic pilot study. Int. J. Ment. Health Syst..

[21] Brann K.L., Boone W.J., Splett J.W., Clemons C., Bidwell S.L. (2020). Development of the school mental health self-efficacy teacher survey using rasch analysis. J. Psychoeduc. Assess..

[22] Han S.S., Weiss B. (2005). Sustainability of teacher implementation of school-based mental health programs. J. Abnorm. Child Psychol..

[23] Ní Chorcora E., Swords L. (2023). Mental health literacy and help-giving responses of Irish primary school teachers. Ir. Educ. Stud..

[24] Rothì D.M., Leavey G., Best R. (2008). On the front-line: teachers as active observers of pupils' mental health. Teach. Teach. Educ..

[25] Franklin C. (2017). The effectiveness of psychosocial interventions delivered by teachers in schools: a systematic review and meta-analysis. Clin. Child Fam. Psychol. Rev..

[26] Li Q. (2020). Teachers' quality of work life and attitudes toward implementing a psychosocial intervention for children affected by parental HIV/AIDS: roles of self-efficacy and burnout. AIDS Care - Psychological and Socio-Medical Aspects of AIDS/HIV.

[27] Ganga N.S., Kutty V.R. (2012). Measuring positive mental health: development of the achutha menon centre positive mental health scale. Asia Pac. J. Publ. Health.

[28] World Health Organization (2018). https://bit.ly/3ljh45s.

[29] Lukat J., Margraf J., Lutz R., van der Veld W.M., Becker E.S. (2016). Psychometric properties of the positive mental health scale (PMH-scale). BMC Psychology.

[30] Lluch-Canut T. (1999). http://diposit.ub.edu/dspace/bitstream/2445/42359/1/E_TESIS.pdf.

[31] Gómez-Acosta A., Vinaccia-Alpi S., Sierra-Barón W. (2020). Psychometric properties of the positive mental health scale in Colombian youth: an exploratory study. Revista CES Psicología.

[32] Garfinkel H. (2016).

[33] Bremm D., da Costa Güllich R.I. (2023). The systematization of experiences as a propeller of research-formation-action in sciences. Investigacoes em Ensino de Ciencias.

[34] Whitney D., Trostem-Bloom A. (2010).

[35] Lluch-Canut T. (2013). Assessing positive mental health in people with chronic physical health problems: correlations with socio-demographic variables and physical health status. BMC Publ. Health.

[36] Deroncele-Acosta A., Brito-Garcías J.G., Sánchez-Trujillo M., de los Ángeles N., Delgado-Nery Y.M., Medina-Zuta P. (2023). Método de modelación teórico-práctica en ciencias sociales. Universidad Y Sociedad.

[37] Hurtado B., Llunch-Canut M.T., Casas I., Sequeira C., Puig M., Roldan J. (2018). Evaluación de la fiabilidad y validez del cuestionario de salud mental positiva en profesores universitarios de enfermería en Cataluña [Evaluation of the Reliability and Validity of the Positive Mental Health Questionnaire in University Nursing Professors in Catalonia]. Revista de Enfermería y Salud Mental.

[38] Neill R.D. (2021).

[39] Flores J., Caqueo-Urízar A., Escobar M., Irarrázaval M. (2022). Well-being and mental health in teachers: the life impact of COVID-19. Int. J. Environ. Res. Publ. Health.

[40] Cohen-Fraade S., Donahue M. (2022). The impact of COVID-19 on teachers' mental health. Journal for Multicultural Education.

[41] Santiago I.S.D. (2023). The impact of the COVID-19 pandemic on the mental health of teachers and its possible risk factors: a systematic review. Int. J. Environ. Res. Publ. Health.

[42] Kush J.M., Badillo-Goicoechea E., Musci R.J., Stuart E.A. (2022). Teachers' mental health during the COVID-19 pandemic. Educ. Res..

[43] Deroncele-Acosta A., Medina-Zuta P., Goñi-Cruz F.F., Ramírez-Garzón M.I., Fernández-Aquino O., Román-Cao E., Montes-Castillo M.M., Gallegos-Santiago E. (2021). Digital competence, role stress and engagement: towards positive mental health in Latin American teachers. Proceedings - 2021 16th Latin American conference on learning technologies. LACLO.

[44] Mérida-López S., Extremera N., Rey L. (2017). Emotion-regulation ability, role stress and teachers' mental health. Occup. Med..

[45] Lanza di Scalea T. (2012). Role stress, role reward, and mental health in a multiethnic sample of midlife women: results from the Study of Women's Health across the Nation (SWAN). J. Wom. Health.

[46] Kelloway E.K., Barling J. (1991). Job characteristics, role stress and mental health. J. Occup. Psychol..

[47] Gan D.Z., McGillivray L., Larsen M.E., Torok M. (2023). Promoting engagement with self‐guided digital therapeutics for mental health: insights from a cross‐sectional survey of end‐users. J. Clin. Psychol..

[48] Bijkerk L.E., Oenema A., Geschwind N., Spigt M. (2023). Measuring engagement with mental health and behavior change interventions: an integrative review of methods and instruments. Int. J. Behav. Med..

[49] Russell K., Rosenbaum S., Varela S., Stanton R., Barnett F. (2023). Fostering community engagement, participation and empowerment for mental health of adults living in rural communities: a systematic review. Rural Rem. Health.

[50] Tucker I., Easton K., Prestwood R. (2023). Digital community assets: investigating the impact of online engagement with arts and peer support groups on mental health during COVID‐19. Sociol. Health Illness.

[51] Wang M.T., Peck S.C. (2013). Adolescent educational success, and mental health vary across school engagement profiles. Dev. Psychol..

[52] Aksunger N. (2023). COVID-19 and mental health in 8 low-and middle-income countries: a prospective cohort study. PLoS Med..

[53] Tomlinson M., Marlow M. (2023). COVID-19 and mental health: building back better or reimagining a new way forward?. PLoS Med..

[54] Deroncele Acosta A., Anaya Lambert Y., López Mustelier R., Santana González Y. (2021). Motivación en empresas de servicios: contribuciones desde la intervención psicosocial. Rev. Venez. Gerenc..

[55] Barbaranelli C., Fida R., Paciello M., Tramontano C. (2018). “Possunt, quia posse videntur”: they can because they think they can. Development and validation of the Work Self-Efficacy scale: evidence from two studies. J. Vocat. Behav..

[56] De Stasio S., Benevene P., Pepe A., Buonomo I., Ragni B., Berenguer C. (2020). The interplay of compassion, subjective happiness and proactive strategies on kindergarten teachers' work engagement and perceived working environment fit. Int. J. Environ. Res. Publ. Health.

[57] Rusu P.P., Colomeischi A.A. (2020). Positivity ratio and well-being among teachers. The mediating role of work engagement. Front. Psychol..

[58] Espinosa-Pérez S., Deroncele-Acosta A., Medina-Zuta P. (2020). Estrategia de intervención psicosocial educativa para el manejo efectivo del estrés de rol en médicos y enfermeras: diagnóstico preliminar y bases epistemológicas [Psychosocial Educational Intervention Strategy for the Effective Management of Role Stress in Physicians and Nurses: preliminary Diagnosis and Epistemological Bases]. Maestro y Sociedad.

[59] Tomás J.M., De Los Santos S., Georgieva S., Enrique S., Fernández I. (2018). Utrecht work engagement scale in Dominican teachers: dimensionality, reliability, and validity. Rev. Psicol. Del Trab. Las Organ..

[60] Zhang M., Bai Y., Li Z. (2020). Effect of resilience on the mental health of special education teachers: moderating effect of teaching barriers. Psychol. Res. Behav. Manag..

[61] Deroncele Acosta A., Medina Zuta P., Gross Tur R. (2020). Gestión de potencialidades formativas en la persona: reflexión epistémica y pautas metodológicas [Managing Formative Potentialities in the Person: epistemic Reflection and Methodological Guidelines]. Universidad y Sociedad.

[62] Seligman M., Csikszentmihalyi M. (2000). Positive psychology: an introduction. Am. Psychol..

[63] Alemán-Saravia A.C., Deroncele-Acosta A. (2021). Proceedings - 2021 16th Latin American Conference on Learning Technologies.

[64] Pînzariu A.I. (2020). An educational paradigm shift: technology-enhanced adaptive and hybrid education. Rev. Appl. Soc. Econ. Res..

[65] Villegas-Ch W., Palacios-Pacheco X., Roman-Cañizares M., Luján-Mora S. (2021). Analysis of educational data in the current state of university learning for the transition to a hybrid education model. Appl. Sci..

[66] Abbasnejad E., Farahani A., Nakhaei A. (2013). The relationship between time management and job stress in teachers of physical education and non-physical education. Adv. Environ. Biol..

[67] Woo B., Evans K., Wang K., Pitt-Catsouphes M. (2021). Online and hybrid education in a social work PhD program. J. Soc. Work. Educ..

[68] Huizinga T., Lohuis A., Zwerver-Bergman J., van der Meer R. (2022). Student and teacher perceptions of community of inquiry in hybrid virtual classrooms. Heliyon.

[69] Fernández-Fernández M., Martínez-Navalón J.-G., Gelashvili V., Román C.P. (2023). The impact of teleworking technostress on satisfaction, anxiety and performance. Heliyon.

[70] Rahiman H.U., Panakaje N., Kulal A., Harinakshi A., Parvin S.M.R. (2023). Perceived academic stress during a pandemic: mediating role of coping strategies. Heliyon.

[71] Thanh N.H., Anh N.N. (2023). The relationship between effective personality and occupational health of lecturers: an empirical assessment in Vietnam. Heliyon.

[72] Krassadaki E., Tsafarakis S., Kapenis V., Matsatsinis N. (2022). The use of ICT during lockdown in higher education and the effects on university instructors. Heliyon.

[73] Deroncele-Acosta A., Palacios-Núñez M.L., Toribio-López A. (2023). Digital transformation and technological innovation on higher education post-COVID-19. Sustainability.

